# Supplementary material for: A new family of cell surface located purine transporters in Microsporidia and related fungal endoparasites
Source: eLife. 2019 Jul 29;8:e47037. doi: 10.7554/eLife.47037 (PMC6699826; doi:10.7554/eLife.47037)
Supplement: Figure 2—source data 1. — SNP analyses was performed on the published RNA-Seq data for T. hominis (Watson et al., 2015). [file elife-47037-fig2-data1.docx]

**Figure 2-source data 1. Single-nucleotide polymorphisms analysis of the RNASeq sequence data across the four ThMFS genes*.**

**ThMFS2 - THOM_1192** (FPKM: 10)

| Genome sequence to Alternative | Number of Reads | Ratio | Codon change | Amino Acid |
| --- | --- | --- | --- | --- |
| A to G | 09:09 | 50%:50% | AAA to AGA | Lys to Arg |
| G to T | 06:09 | 40%:60% | GTC to GTT | Val |
| A to G | 13:02 | 87%:13% | TTA to TTT | Leu to Phe |
| A to G | 17:08 | 68%:32% | GTA to GTG | Val |
| G to A | 13:04 | 76%:24% | GTA to ATA | Val to Ile |
| T to C | 11:09 | 45%:55% | GGT to GGC | Gly |

**ThMFS3 - THOM_1681** (FPKM: 20)

| A to G | 07:05 | 58%:42% | CTA to CTG | Leu |
| --- | --- | --- | --- | --- |
| C to T | 17:09 | 65%:35% | CTG to TTG | Leu |

**ThMFS4 - THOM_3170** (FPKM: 23)

| A/C/T | 4:4:11 | 21%:21%:58% | AAT / AAC/ AAA | Asn/Asn/Lys |
| --- | --- | --- | --- | --- |

**ThMFS1 - THOM_0963** (FPKM: 389)

| G to A | 117:5 | 96%:4% | AAG to AAA | Lys |
| --- | --- | --- | --- | --- |

*RNA-Seq data from Watson et al. (2015)[17].

Entries are listed by decreasing level of SNP diversity with ThMFS1 and ThMFS3 characterised by exclusively synonymous SNPs.
