# Supplementary material for: A new family of cell surface located purine transporters in Microsporidia and related fungal endoparasites
Source: eLife. 2019 Jul 29;8:e47037. doi: 10.7554/eLife.47037 (PMC6699826; doi:10.7554/eLife.47037)
Supplement: Transparent reporting form [file elife-47037-transrepform.docx]

***eLife’s* transparent reporting form**

We encourage authors to provide detailed information *within their submission* to facilitate the interpretation and replication of experiments. Authors can upload supporting documentation to indicate the use of appropriate reporting guidelines for health-related research (see [EQUATOR Network](http://www.equator-network.org/%20)), life science research (see the [BioSharing Information Resource](https://biosharing.org/)), or the [ARRIVE guidelines](http://www.plosbiology.org/article/info:doi/10.1371/journal.pbio.1000412) for reporting work involving animal research. Where applicable, authors should refer to any relevant reporting standards documents in this form.

If you have any questions, please consult our Journal Policies and/or contact us: [editorial@elifesciences.org](mailto:editorial@elifesciences.org).

**Sample-size estimation**

- You should state whether an appropriate sample size was computed when the study was being designed
- You should state the statistical method of sample size computation and any required assumptions
- If no explicit power analysis was used, you should describe how you decided what sample (replicate) size (number) to use

Please outline where this information can be found within the submission (e.g., sections or figure legends), or explain why this information doesn’t apply to your submission:

**Replicates**

- You should report how often each experiment was performed
- You should include a definition of biological versus technical replication
- The data obtained should be provided and sufficient information should be provided to indicate the number of independent biological and/or technical replicates
- If you encountered any outliers, you should describe how these were handled
- Criteria for exclusion/inclusion of data should be clearly stated
- High-throughput sequence data should be uploaded before submission, with a private link for reviewers provided (these are available from both GEO and ArrayExpress)

Please outline where this information can be found within the submission (e.g., sections or figure legends), or explain why this information doesn’t apply to your submission:

For the RNA-Seq data, all relevant information is provided in the “Materials and Methods” section under the subheading “RNA-Seq data generation and analyses”. Previous studies (in triplicate) indicated that the variation between independent replicates for this experimental design was low (Watson et al., 2015), so a sample size of N=2 was used for this study. The RNA-Seq data used to generate Fig. 2A and 2B are tabulated in the Fig. 2-Source data 2.

For transport assays all relevant information about biological replicates (different E. coli clones expressing a given transporter or control clones transformed with expression vector without cloned transporter gene) are provided in the figure legends. The sample size for all transport experiment is N=3 and sample variation is represented by standard deviations (Fig. 3, Fig. 4 and Fig. 5). Several independent experiments were performed using different bacteria colonies to ensure the different transport data were robust. In none of the presented data were values identified as outliers. For transport data of nucleoside and nucleotides the raw transport data are tabulated in the respective Source data files (Fig. 3-source data 1; Fig. 4-Source data 1; Fig. 5-Source data 1).

For the quantification of IFA signal the raw quantification values are tabulated in the Fig. 2-Source data 3 (N=3-6 depending on the quantified signal).

**Statistical reporting**

- Statistical analysis methods should be described and justified
- Raw data should be presented in figures whenever informative to do so (typically when N per group is less than 10)
- For each experiment, you should identify the statistical tests used, exact values of N, definitions of center, methods of multiple test correction, and dispersion and precision measures (e.g., mean, median, SD, SEM, confidence intervals; and, for the major substantive results, a measure of effect size (e.g., Pearson's r, Cohen's d)
- Report exact p-values wherever possible alongside the summary statistics and 95% confidence intervals. These should be reported for all key questions and not only when the p-value is less than 0.05.

Please outline where this information can be found within the submission (e.g., sections or figure legends), or explain why this information doesn’t apply to your submission:

For the phylogenetic analyses ultrafast bootstrap support value for branches were generated with 1000 samples. This information is provided in the figure legends (Fig. 1, and associated figure supplement 1 and 2) and the Materials and Methods section. According to the IQ-TREE manual one should only start to believe in a clade if its support is ≥95%.

For the RNA-Seq data illustrated in Figure 2A the mean values are shown along the raw values of both replicates. All RNA-Seq values discussed in the manuscript (for the ThMFS and ThNTT genes) are also listed in the Fig. 2-Source data 2.

(For large datasets, or papers with a very large number of statistical tests, you may upload a single table file with tests, Ns, etc., with reference to sections in the manuscript.)

**Group allocation**

- Indicate how samples were allocated into experimental groups (in the case of clinical studies, please specify allocation to treatment method); if randomization was used, please also state if restricted randomization was applied
- Indicate if masking was used during group allocation, data collection and/or data analysis

Please outline where this information can be found within the submission (e.g., sections or figure legends), or explain why this information doesn’t apply to your submission:

**Additional data files (“source data”)**

- We encourage you to upload relevant additional data files, such as numerical data that are represented as a graph in a figure, or as a summary table
- Where provided, these should be in the most useful format, and they can be uploaded as “Source data” files linked to a main figure or table
- Include model definition files including the full list of parameters used
- Include code used for data analysis (e.g., R, MatLab)
- Avoid stating that data files are “available upon request”

Please indicate the figures or tables for which source data files have been provided:

For the RNA-Seq data illustrated in Figure 2A the mean values are graphed with the raw values of replicates and all raw values are also listed in the Fig. 2-Source data 2. For transport data shown in Fig. 3 and Fig. 4 the corresponding raw transport data are tabulated in the respectively Fig. 3-Source data 1 and Fig. 4-Source data 1.

The transport data in Fig. 5 are illustrated as the proportion (%) of transport observed in samples treated with CCCP compared to respective untreated controls. The full transport source data for this experiment are plotted in Fig. 5-figure supplement 1 and the raw data are tabulated in Fig. 5-Source data 1.
